# Anti-Inflammatory Activity of Pequi Oil (*Caryocar brasiliense*): A Systematic Review

**DOI:** 10.3390/ph17010011

**Published:** 2023-12-21

**Authors:** Vitória R. P. Silva, Andréia C. Pinheiro, Alicia S. Ombredane, Natália Ornelas Martins, Glécia V. S. Luz, Marcella L. B. Carneiro, Graziella A. Joanitti

**Affiliations:** 1 Laboratory of Bioactive Compounds and Nanobiotechnology (LBCNano), Faculty of Ceilandia, University of Brasilia, Centro Metropolitano, Ceilândia Sul, Brasilia 72220-275, DF, Brazil; vitoriarpsilva@gmail.com (V.R.P.S.); andreiacp@gmail.com (A.C.P.); aliciaombredane@gmail.com (A.S.O.); nataliaornelas8@gmail.com (N.O.M.); marbretas@gmail.com (M.L.B.C.); 2 Post-Graduate Program in Pharmaceuticals Sciences, Faculty of Health Sciences, University of Brasilia, Campus Darcy Ribeiro, Brasilia 70910-900, DF, Brazil; 3 Post-Graduation Program in Nanoscience and Nanobiotechnology, Institute of Biological Sciences, University of Brasilia, Campus Darcy Ribeiro, Brasilia 70910-900, DF, Brazil; 4 Health Technology Assessment Center-NATS/UnB, University of Brasilia, Campus Darcy Ribeiro, Brasilia 70910-900, DF, Brazil; gleciavs@gmail.com; 5 Post-Graduation Program in Biomedical Engineering, Faculty of Gama, University of Brasilia, Brasilia 72444-240, DF, Brazil

**Keywords:** anti-inflammatory, pequi oil, *Caryocar brasiliensis*, natural products, nanoemulsions, systematic review

## Abstract

Disorders in the inflammatory process underlie the pathogenesis of numerous diseases. The utilization of natural products as anti-inflammatory agents is a well-established approach in both traditional medicine and scientific research, with studies consistently demonstrating their efficacy in managing inflammatory conditions. Pequi oil, derived from *Caryocar brasiliense*, is a rich source of bioactive compounds including fatty acids and carotenoids, which exhibit immunomodulatory potential. This systematic review aims to comprehensively summarize the scientific evidence regarding the anti-inflammatory activity of pequi oil. Extensive literature searches were conducted across prominent databases (Scopus, BVS, CINAHL, Cochrane, LILACS, Embase, MEDLINE, ProQuest, PubMed, FSTA, ScienceDirect, and Web of Science). Studies evaluating the immunomodulatory activity of crude pequi oil using in vitro, in vivo models, or clinical trials were included. Out of the 438 articles identified, 10 met the stringent inclusion criteria. These studies collectively elucidate the potential of pequi oil to modulate gene expression, regulate circulating levels of pro- and anti-inflammatory mediators, and mitigate oxidative stress, immune cell migration, and cardinal signs of inflammation. Moreover, negligible to no toxicity of pequi oil was observed across the diverse evaluated models. Notably, variations in the chemical profile of the oil were noted, depending on the extraction methodology and geographical origin. This systematic review strongly supports the utility of pequi oil in controlling the inflammatory process. However, further comparative studies involving oils obtained via different methods and sourced from various regions are warranted to reinforce our understanding of its effectiveness and safety.

## 1. Introduction

The inflammatory process is a complex mechanism orchestrated by the immune system in response to harmful stimuli, such as infections and tissue injuries. Its primary objective is to restore the organism to a state of homeostasis. However, in cases of disorder, this mechanism can become exaggerated, rendering it ineffective in achieving its intended goal. Therefore, controlling the inflammatory process through anti-inflammatory mediators is a crucial therapeutic approach for treating several diseases characterized by uncontrolled inflammation [1].

The rich diversity of plant species in Brazil establishes Brazilian biodiversity as a significant reservoir of natural resources with notable therapeutic potential [2,3]. While around 55,000 species have been cataloged in Brazil, only about 1100 have undergone scientific scrutiny regarding their therapeutic properties. This scenario highlights the vast untapped potential within Brazilian biodiversity, which can serve as the foundational material for pioneering pharmaceutical product development [4,5].

Pequi is the fruit of the *Caryocar brasiliense* tree, native to the Brazilian Cerrado. The pulp of the pequi yields an oil widely utilized in both culinary practices and traditional medicine. The therapeutic benefits of pequi oil stem from the presence of various bioactive compounds, including fatty acids and carotenoids. These molecules play a significant role in interacting with inflammation-promoting mechanisms. They have the capacity to modulate inflammatory responses by regulating the release of cytokines and the activity of immune system cells involved in the inflammatory cascade. As a result, anti-inflammatory effects have been observed in in vitro, in vivo, and clinical models [6].

Despite the growing number of publications on the anti-inflammatory activity of pequi oil, to the best of our knowledge, no systematic review has yet been conducted to comprehensively analyze the findings of these studies. Given the crucial role of this type of review as a tool for analyzing and synthesizing scientific evidence, this study aims to systematically compile the information available in the literature regarding the anti-inflammatory properties of pequi oil extracted from the fruit of the species *C. brasiliensis*. The objective is to provide a comprehensive overview of the anti-inflammatory effectiveness and toxicity of pequi oil, including considerations on the impacts of extraction methods, chemical characterization, and mechanisms of immunomodulatory action across various inflammatory models. The discussion of these pertinent findings can serve as a guide for future scientific investigations, forming the basis for advancing the clinical applications of pequi oil in the treatment of inflammatory pathologies.

## 2. Material and Methods

### 2.1. Protocol and Registration

The study was conducted in accordance with the criteria outlined in the PRISMA checklist (Preferred Reporting Items for Systematic Reviews and Meta-Analyses) [7]. The protocol for this systematic review was registered in the International Prospective Register of Systematic Reviews (PROSPERO) [8] under the registration number CRD42023335323.

### 2.2. Eligibility Criteria

#### 2.2.1. Inclusion Criteria

The inclusion criteria for this systematic review were established based on the PICO framework, representing population, intervention, comparators, and outcome [9]. We included studies that assessed the anti-inflammatory activity (outcome) of crude oil extracted from the fruit of the *Caryocar brasiliense* species (pequi) (intervention), compared with negative controls (comparators), across in vitro, in vivo, and clinical trial models (population).

#### 2.2.2. Exclusion Criteria

The following criteria were used to exclude articles from this systematic review: (i) pequi oil derived from species other than *Caryocar brasiliense*; (ii) fractionated pequi oil or other isolated compounds from *Caryocar brasiliense*; (iii) studies that did not evaluate anti-inflammatory activity in in vitro, in vivo, or clinical test models; and (iv) review articles, book chapters, theses, letters, personal opinions, conference abstracts, and patents.

### 2.3. Information Sources and Search Strategy

The literature search was conducted by creating distinct search strings tailored to each respective bibliographic database consulted, which included CINAHL, Cochrane, LILACS, Embase, FSTA, MEDLINE, ProQuest, BSV regional portal, PubMed, ScienceDirect, Scopus, and Web of Science (Table S1). This search was performed in 2022, without imposing any language restrictions or publication period limitations. Duplicate references were eliminated using reference management software (Mendeley^®^ version 1.19.4).

### 2.4. Study Selection

The article selection process was conducted in two phases to verify the suitability of each study according to the inclusion and exclusion criteria. In phase one, two authors (V.R.P.S. and A.C.P.) assessed the title and abstract of all studies identified in the search results. In phase two, the authors were organized into four pairs (V.R.P. and A.C.P.; A.S.O. and N.O.M.; M.L.B.C. and G.A.J.) and independently examined the full text of the studies included in phase one, excluding those that did not meet the inclusion criteria (Table S2). Following the completion of the selection phase, relevant information from the included studies was independently extracted by the pairs. Any disagreements between the pairs were resolved through consultation with a third author.

### 2.5. Quality Analysis in Individual Studies

The quality analysis of the studies included in this review was conducted using the ARRIVE Guideline 2.0 [10], adapted to include 21 questions (see Table S3). These questions were independently answered by the pairs, with any disagreements resolved by a third reviewer. Responses of HIGH indicated high quality, LOW indicated low quality, UNCLEAR indicated that quality measurement was not possible, NO INFORMATION indicated that the information was not found in the article, and NOT APPLICABLE if the type of study does not apply to the question.

## 3. Results and Discussion

### 3.1. Study Selection

On 29 January 2023, utilizing the search string “(ALL ((pequi OR ‘pequi oil’) AND caryocar))” in the Scopus database, we identified a growing number of studies related to pequi, totaling 1490 publications. Intriguingly, when we refined the search to specifically focus on studies involving pequi oil from the species *Caryocar brasiliensis* using the search string “(ALL (‘pequi oil’ AND ‘*Caryocar brasiliensis*’))”, applied to the Scopus database on the same date, we found 46 published studies. Notably, 82.6% of these were authored by groups of Brazilian researchers.

**Figure 1:**
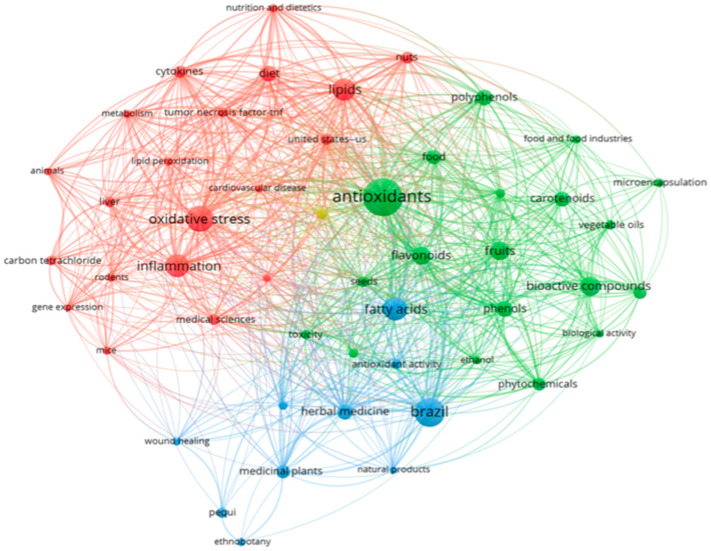
Co-occurrence and connectivity of terms found in “Titles, Keywords and Abstracts” of retrieved publications of the databases. Analysis using VOSviewer software 1.6.18 (2022); minimum 10×; “full count” configuration. Source: SCOPUS, BVS, CINAHL, Cochrane, Lilacs, Embase, ProQuest, PubMed, ScienceDirect, Web of Science (2023).

When associating pequi oil with inflammation using the search string described in Table S1, a total of 377 publications were retrieved from various databases (SCOPUS, BVS, CINAHL, Cochrane, Lilacs, Embase, ProQuest, PubMed, ScienceDirect, and Web of Science) up until January 2023. In terms of the references within these documents, Figure 1 illustrates the co-occurrence of terms (appearing at least 10 times, configured with ‘full count’) in their titles, keywords, and abstracts. The analysis presented in Figure 1 also provides insights into the most frequently co-occurring terms, which are represented by larger spheres. Notably, the academic community’s focus encompasses key topics such as oxidative stress, antioxidants, flavonoids, inflammation, fatty acids, and lipids.

**Figure 2:**
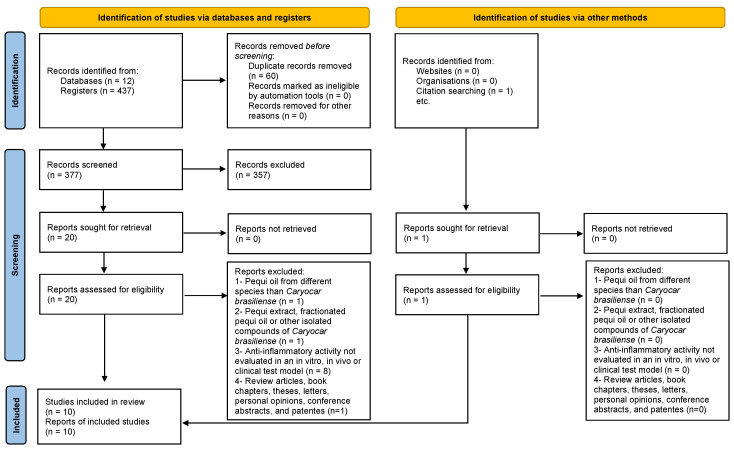
Flow diagram of the literature search and selection criteria from PRISMA [11].

In this systematic review, as depicted in Figure 2, a total of 437 studies were identified across various databases (84 from Scopus, 64 from ScienceDirect, 3 from LILACS, 31 from Embase, 22 from MEDLINE, 25 from BSV Regional Portal, 5 from PubMed, 22 from Web of Science, 21 from CINAHL, 12 from FSTA, and 142 from ProQuest). After removing duplicates, 377 studies remained. Subsequently, an evaluation of the ‘title and abstract’ led to the exclusion of 357 studies. The remaining 20 articles underwent a full-text review. Through this process, 11 articles were excluded based on the predefined criteria (see Table S2). Additionally, one article was included in this review through citation searching. Ultimately, 10 articles were analyzed in this systematic review.

### 3.2. Characteristics of the Included Studies (n = 10)

All the included studies were research articles that assessed the anti-inflammatory effects of pequi oil. The main characteristics of these studies are summarized in Table 1.

Interestingly, all the selected studies were conducted in Brazil and were published in English between 2009 and 2021.

The pequi oil used in these studies was primarily extracted through solvent methods (*n* = 4) [13,15,16,20], boiling (*n* = 1) [6], cold-pressed/centrifugation (*n* = 4) [6,14,17,19], and in two cases, the extraction method was not specified [12,18]. Additionally, some studies assessed the oil’s characteristics by analyzing the lipid profile (*n* = 9) and other metabolites (*n* = 7).

The anti-inflammatory effect of pequi oil (PO) was assessed in both in vitro (*n* = 2) and in vivo (*n* = 6) studies, utilizing various models involving inflammatory markers (see Table 1). These models included atherosclerosis (*n* = 1) [12], ulcerative colitis (*n* = 1) [14], pleurisy (*n* = 1) [14], pulmonary inflammation (*n* = 1) [13], paw edema (*n* = 1) [14], as well as assessments of antioxidant activity and its impact on inflammation (*n* = 2) [6,19]. In clinical studies (*n* = 4), the effects of oral supplementation with pequi oil on inflammatory markers, such as lipid peroxidation, were compared among healthy volunteers (runners) post-races and patients with systemic lupus erythematosus (see Table 1) [13,15,16,17,20].

In terms of experimental design, both in vitro and in vivo studies displayed heterogeneity with regards to the animal model used, the duration of the experiment, and the treatment dosage. One in vitro study assessed the antioxidant activity of pequi oil using the DPPH assay [19], while another investigated ROS production in a primary culture of peritoneal resident macrophages from animals that were orally supplemented with 7% pequi oil for 2 weeks [12]. Among the in vivo studies, 84% utilized mice as the animal model (*n* = 5), while 16% employed rats (*n* = 1) [6]. The mouse strains included C57BL/6 (*n* = 2; both wild-type and genetically modified), A/J (*n* = 1), and Swiss (*n* = 2). The Wistar strain was used for the study with rats. In terms of rodent age, the effects of pequi oil were evaluated in mice aged 6–8 weeks (*n* = 2) [12,14], and 6–12 months (*n* = 1) [19], which are roughly equivalent to adolescent and elderly humans, respectively [21]. The rodent age in three in vivo studies was not specified [6,13,18].

In all six in vivo studies, oral administration was the chosen route. Among these, two studies incorporated pequi oil into the diet mixture [12,18]. Pequi oil was administered either on a short-term basis (ranging from 1 to 18 h; *n* = 2) or over a longer period (spanning from 2 weeks to 6 weeks; *n* = 4). The doses of pequi oil used in the treatments varied, ranging approximately from 20 mg to 1000 mg per kg (*n* = 3). In one study, the provided dose ranged from 3 to 6 mL/kg. However, this dose was not converted to “mg/kg” due to the absence of additional details regarding oil density [6]. In two studies, it was not feasible to precisely calculate the administered dose since the oil was mixed with the diet and there was no available information on the average chow consumption per experimental group [12,18].

In all four clinical studies, a daily oral administration of 400 mg of pequi oil was adopted, with treatment durations of 14 days in three studies and 60 days in one study (see Table 1). The study participants included patients with systemic lupus erythematosus (*n* = 1) and healthy individuals with a minimum running performance of 4000 m, comprising both men and women aged between 15 and 67 years old (*n* = 3).

### 3.3. Quality of Individual Studies

**Figure 3:**
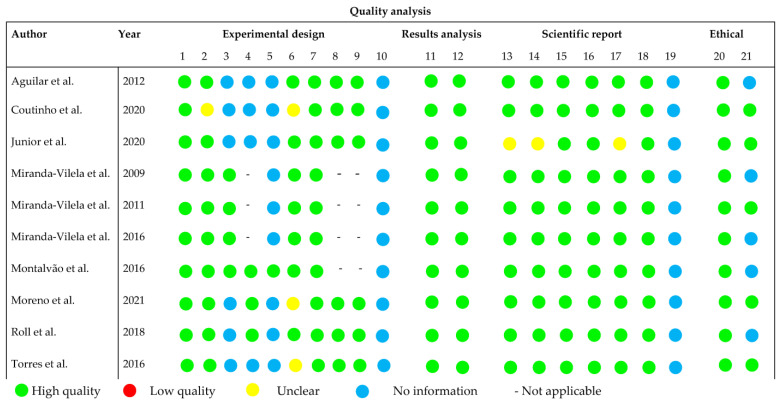
Overall quality of the selected studies [6,8,12,13,14,15,16,17,18,19]. Detailed description of the evaluated parameters is found in Table S3.

The quality assessment was conducted based on the ARRIVE guidelines for all the included studies (see Figure 3), with criteria that were not clearly reported or had incomplete information being classified as ‘unclear’. Most studies demonstrated a well-executed study design, and a number of articles explicitly mentioned the presence of control groups (*n* = 10), route of administration (*n* = 10), duration of treatment (*n* = 10), and dosage of pequi oil (*n* = 9). In both in vivo and clinical studies, ethical committee approval was clearly reported in all cases. However, most studies did not provide clear information regarding blinding (*n* = 9), allocation/randomization (*n* = 4), and the age of the evaluated rodents (*n* = 3), which are crucial factors for quality assessment.

### 3.4. Physiology and Control of the Inflammatory Process

Inflammation is a complex process characterized by the dynamic action of molecules and cells of the immune system. Its aim is to reestablish homeostasis in a tissue site that has been injured or invaded by pathogens [22]. The orchestration of inflammation occurs through an orderly balance of the production of pro- and anti-inflammatory mediators, responsible for the initiation and subsequent cessation of the inflammatory response [23]. These mediators act via paracrine, autocrine, and endocrine signals. They regulate inflammation and induce cellular changes including modulation of gene expression, redirection of energy metabolism, activation of biosynthesis pathways, cytoskeletal rearrangement, and release of cytoplasmic granules [24,25,26].

The activation of the inflammatory process occurs through the recognition of molecular patterns associated with pathogens and damage (known as PAMPs and DAMPs, respectively) by pattern recognition receptors expressed in cells of the immune system, primarily macrophages, dendritic cells, and mast cells [24,27]. The recognition of these patterns stimulates the release of pro-inflammatory signaling molecules such as MCP-1, TNFα, IL-6, IL-1β, and arachidonic acid-derived eicosanoids (including prostaglandins and leukotrienes). These molecules promote the activation and recruitment of new leukocytes and lymphocytes to the inflammatory site, thereby integrating the innate and adaptive immune responses [23,27].

The inflammatory microenvironment is beneficial when formed in acute situations in response to harmful stimuli, promoting the eradication of infectious agents and preparing the injured tissue for remodeling [24]. However, the perpetuation of this process and its systemic establishment can impact the functioning of various tissues, leading to diseases of an inflammatory nature, such as Crohn’s disease, Alzheimer’s disease, atherosclerosis, acne, heart attacks, carcinogenesis, and other complications [28].

The resolution of inflammation is an active process mediated by the production of anti-inflammatory factors that signify the return of tissue to its physiological state [22]. Macrophages play a central role in this process owing to their high stimulus-dependent plasticity [24]. When signaled by regulatory cytokines such as IL-4, IL-10, and IL-13, macrophages phagocytize cellular debris and activate metabolic pathways related to phosphorylative oxidation. They also produce lipid anti-inflammatory mediators derived from ω-3 polyunsaturated fatty acids, including resolvins, protectins, and maresins. These mediators prevent the chronicity of the inflammatory process by limiting the tissue migration of neutrophils and inhibiting the release of pro-inflammatory cytokines and eicosanoids [22,24,27,29,30].

In addition to the physiological resolution process, inflammation can be halted through the use of anti-inflammatory drugs, which act by directly inhibiting the genesis and maintenance pathways of inflammation [22]. Despite their effectiveness in curtailing inflammation, most of these drugs lead to severe adverse effects due to their interaction with components crucial for the physiological functioning of certain tissues [31]. For instance, non-steroidal anti-inflammatory drugs (NSAIDs) operate by inhibiting the cyclooxygenase (COX) enzyme, which is responsible for the production of prostaglandins. However, this inhibition directly impacts the enzyme’s roles in physiological functions related to hemostasis, gastric cytoprotection, among others. Consequently, this can result in adverse effects such as gastric lesions, hypertension, nephropathies, and others [31]. Therefore, the discovery of new molecules capable of reducing inflammation without affecting the homeostasis of healthy tissues is of great relevance.

Brazilian biodiversity significantly contributes to the discovery of new molecules with therapeutic potential [32]. Among the sources under study, fixed oils extracted from plant sources have garnered considerable attention for their anti-inflammatory properties [33]. Numerous studies have assessed the capacity of pequi oil to modulate inflammation through experimental models, both in vitro and in vivo, along with clinical trials investigating its impact on clinical, cellular, and molecular aspects of inflammation, as well as its role in the progression of various inflammatory diseases.

### 3.5. Anti-Inflammatory Effect of Pequi Oil in Inflammatory Pathologies

#### 3.5.1. Atherosclerosis

Atherosclerosis is a disease characterized by the excessive deposition of lipoproteins containing apolipoprotein B in the intima of the arterial wall, resulting from the loss of endothelial integrity due to stresses on blood flow [34]. Changes in the structure of the endothelium and the oxidation of low-density lipoproteins (oxLDL) activate both innate and adaptive immune responses, giving rise to a chronic inflammatory condition responsible for impairments in the functioning of the cardiovascular system [35]. The significant role of inflammatory mechanisms in the genesis and progression of atherosclerosis underscores the potential utility of anti-inflammatory therapies as approaches to prevent cardiovascular events [36].

Pequi oil is composed of both saturated and unsaturated fatty acids, which play significant roles in atherogenesis, particularly concerning the inflammatory process, vascular endothelial integrity, and the promotion of dyslipidemia [12,34]. The interaction of these fatty acids with the plasma membrane of endothelial cells affects their fluidity and leads to an increase in the expression of adhesion molecules crucial for initiating the inflammatory response at atherogenic sites [34].

Despite the atherogenic effects promoted by the fatty acids present in pequi oil, Aguilar (2012) demonstrated a reduction in the number of atherosclerotic lesions in the aortas of female C57BL/6 mice, which were LDL receptor-deficient and treated with pequi oil [12]. This effect may be attributed to the presence of secondary metabolites in the oil composition, such as carotenoids, tocopherols, and phenolic compounds, exhibiting electron-dense regions capable of neutralizing free radicals and thereby attenuating the oxidative stress produced during the inflammatory process. Additionally, these compounds play a crucial role in decreasing oxLDL levels and its immunogenicity, as observed in the study [6,12]. However, in the same study by Aguilar, the antioxidant effect of pequi oil was unable to counterbalance its atherogenic mechanisms in the aortic root of mice, resulting in an increase in the number of atherosclerotic lesions in this region. It is noteworthy that this region is anatomically predisposed to the development of atheroma plaques due to the high turbulence of blood flow and the presence of myeloid cells in the intimal arterial layer [12,37].

Supporting the atheroprotective findings of pequi oil, clinical studies involving runners conducted by Miranda-Vilela (2009, 2011, 2016) demonstrated that supplementation with the oil was effective in inhibiting platelet activation, as evidenced by the reduced platelet count and mean platelet volume. Interestingly, contrary to the anticipated impacts on the lipid profile following oil ingestion, volunteers exhibited reduced levels of LDL, VLDL, total cholesterol, triglycerides, and an increase in HDL. These atherogenic effects of pequi oil were also linked to its modulatory influence on polymorphisms in components associated with endogenous antioxidant mechanisms and the regulation of cardiovascular function [15,16,20].

#### 3.5.2. Ulcerative Colitis

Ulcerative colitis (UC) is a chronic idiopathic inflammatory disorder affecting the colon. This condition leads to persistent mucosal inflammation spanning from the rectum to the more proximal colon, with varying lengths, and is characterized by a recurrent and remitting course. Classic symptoms include bloody diarrhea with or without mucus, rectal urgency, and variable degrees of abdominal pain. UC is identified by features such as erythema, loss of the normal vascular pattern, granularity, erosions, friability, bleeding, and ulcerations, often with a distinct demarcation between inflamed and non-inflamed bowel [38].

The interaction between microbiota, intestinal epithelium, and the immune system is a pivotal factor in the pathophysiology of UC. This interplay can lead to alterations in the cell profile, resulting in an augmented production of inflammatory cytokines and reactive oxygen species. Additionally, the immune system can instigate intestinal inflammation through both innate and adaptive responses. Recent evidence suggests that various factors may disrupt intestinal homeostasis, presenting future challenges in the development of novel therapies for UC [39,40].

Foods with immune-modulatory properties could complement pharmacological treatments for ulcerative colitis by promoting the maintenance of intestinal integrity. In this context, one prominent nutraceutical food is pequi oil, which contains substantial amounts of immunomodulatory monounsaturated fatty acids, particularly oleic acid, and carotenoids. Monounsaturated fatty acids (MUFA) may mitigate the intensity of the inflammatory response by reducing cytokine secretion and proinflammatory plasma proteins. Carotenoids, widely recognized for their antioxidant activity, can mitigate the harmful effects of elevated levels of free radicals by inducing a modulation of the immune system [18].

Previous studies have already demonstrated the biological effects of pequi oil, including its antioxidant and anti-inflammatory properties. Moreno (2011) assessed the impact of pequi oil on components of the intestinal immune response in mice with dextran sulfate sodium (DSS)-induced ulcerative colitis. The study showed that the consumption of pequi oil contributed to the regulation of the immune response and improved the clinical and histological indicators in the mouse model of UC. They observed a reduction in cytotoxicity and other inflammatory markers, along with the stimulation of regulatory cells, which preserved the mucus-producing cells. This study provided new insights into the importance of regular pequi oil intake for a more favorable prognosis in acute ulcerative colitis [18].

#### 3.5.3. Pulmonary Inflammation, Local Inflammation, and Nociception

Several airway diseases are associated with the inflammation of lung structures. For instance, acute respiratory distress syndrome (ARDS) and acute lung injury (ALI) are inflammatory pulmonary disorders where damage to the alveolar–capillary barrier leads to the release of neutrophil granules and oxidative injury [41,42]. Additionally, some of the causes of pleurisy, a condition where the layers separating the lungs from the chest are injured, are also related to inflammation [43]. Excessive and chronic inflammation in the lungs can be life-threatening, given the vital role of this organ.

Interestingly, a single oral dose of pequi oil (1000 mg/kg) administered one hour prior to pleurisy induction demonstrated a significant reduction in leukocyte migration to the pleural exudate [14]. Similar effects were reported by Coutinho (2020), where a single oral treatment with pequi oil a few hours before inducing pulmonary inflammation resulted in a significant decrease in the migration of leukocytes and neutrophils to the lung. It is worth noting that more pronounced anti-inflammatory effects were observed when pequi oil was administered within nanocarriers [13]. The advantages of utilizing nanostructures for delivering natural oils are explored in the section titled “Nanostructured pequi oil”.

The fatty acid composition of pequi oil plays a crucial role in its anti-inflammatory effects. Palmitic acid is known to induce pro-inflammatory effects on immune cells [44]. Conversely, the effects of oleic acid, one of the main fatty acids in pequi oil, on lung inflammation are a subject of debate. While several studies indicate that intravenous injection of oleic acid can lead to lung injury and inflammation [45], research evaluating the oral intake of oleic acid provides evidence of its anti-inflammatory role in ARDS and other respiratory diseases [46,47], supporting the findings of Coutinho (2020) and Junior et al. (2020). It is worth noting that inflammation in the lungs and their structures often involves oxidative stress associated with tissue damage [41,42]. In this context, the antioxidant bioactive compounds found in pequi oil, such as carotenoids and phenolic compounds [6,48], may also contribute to the observed anti-inflammatory effects in the models of airway diseases examined by Junior and colleagues (2020) and Coutinho and colleagues (2020) [13,14].

Pequi oil has been noted in the literature for its anti-inflammatory and analgesic properties. Animals treated with pequi oil, and subsequently subjected to carrageenan-induced lower limb edema, experienced a reduction in swelling of up to 60%, and a decrease in pain of up to 95% when formalin was used to induce pain [14]. This reduction is attributed to the anti-inflammatory activity provided by flavonoids and terpenoids present in pequi oil, which potentially counteract the effects of the release of inflammatory cytokines. Additionally, other studies highlight the anti-inflammatory potential of oleic and palmitic fatty acids, which are abundant in pequi oil. These fatty acids have been examined for their capacity to inhibit the inflammatory activation cascade via the metabolites of arachidonic acid, thereby modulating the inflammatory response. This may elucidate the observed reduction in carrageenan-induced hind limb edema in animals previously treated with pequi oil [49].

#### 3.5.4. Autoimmune Diseases

Autoimmune diseases are initiated by the immune system’s activation against self-antigens, leading to an immune response that results in damage to healthy tissues. Systemic lupus erythematosus (SLE) is a chronic autoimmune disease characterized by heightened activity of B- and T-lymphocytes, along with extensive production of reactive autoantibodies, predominantly antinuclear. These antibodies form complexes that are deposited in tissues, contributing to the onset of various clinical manifestations [50]. Additionally, individuals with SLE exhibit a higher incidence of DNA damage, which may be linked to the oxidative stress induced by the inflammatory process [17].

The oral administration of 400 mg of pequi oil in patients with SLE did not demonstrate significant effects in reducing DNA damage. Additionally, these patients exhibited resistance to the oil’s effects on the lipid profile; this is in contrast to other studies involving runners who received pequi oil supplementation at the same dose [17,20]. These conflicting results may be attributed to differences in oil extraction methods between the two studies. In the study with SLE patients, the oil was extracted through cold-pressing, while in the study with runners, extraction was performed using an organic solvent capable of extracting higher concentrations of antioxidant components, such as carotenoids [16,17,20]. Furthermore, the heightened activity of the immune system in SLE induces alterations in lipid metabolism that may influence sensitivity to the effects of pequi oil [51].

Despite these findings, the ingestion of pequi oil by SLE patients was able to reduce hs-CRP levels [17]. However, it is important to note that while CRP is a classic marker of inflammation, this does not universally apply to all types of inflammation. In the case of SLE, the inflammatory response is characterized by type 1 interferon cytokines. In the presence of an exacerbated inflammatory condition, with high production of IL-6, CRP levels may remain moderate. Therefore, it cannot be conclusively stated that there is a direct correlation between reduced levels of hs-CRP and the anti-inflammatory activity of pequi oil [52]. Furthermore, CRP may assist in the elimination of antibody complexes formed in SLE by activating the complement system and stimulating phagocytosis. This suggests that it may have potential beneficial effects in patients with SLE [52].

#### 3.5.5. Antioxidant Activity of Pequi Oil and Impacts on Inflammatory Processes

Oxidative stress is defined as an imbalance between the circulating levels of reactive oxygen species (ROS) and the capacity of endogenous antioxidant mechanisms to neutralize them [53]. These free radicals exhibit instability due to the presence of unpaired electrons, enabling them to react with crucial cellular macromolecules, thereby compromising cell viability [54]. Additionally, ROS are released during inflammation, where they serve vital roles as mediators of cytokine and NF-kB signal transduction and facilitate the elimination of infectious agents through oxidative attacks. However, it is important to note that excessive inflammation is associated with promoting oxidative stress, and notably, elevated levels of ROS can also activate the inflammatory process [54,55].

Antioxidants are molecules capable of being oxidized by free radicals, thus reducing their circulating levels. Pequi oil is rich in antioxidants like carotenoids, vitamin E, and polyphenols. Due to this, numerous studies have investigated the use of the oil in preventing cell damage from oxidative stress [6,19,48,56]. In a study conducted by Vale (2019), it was demonstrated that supplementation with pequi oil could attenuate the oxidative stress induced by physical exercise in rats. This led to the preservation of liver tissue integrity, a reduction in ROS levels, and a decrease in signs of inflammation such as lymphocyte infiltration [56].

In addition to directly engaging in antioxidant reactions like electron transfer, hydrogen atom abstraction, and incorporation of free radicals into the molecular structure, pequi oil also stimulates endogenous antioxidant mechanisms. This was demonstrated in a study by Torres (2016) involving rats, where oil supplementation led to increased activity of GR, GPX, and SOD enzymes, which play pivotal roles in the detoxification of free radicals [6,56]. The results of the DPPH assay further support the observed antioxidant activity of the oil in vivo, as a dose of 26.26 mg/mL of oil reduced DPPH levels by 50% [19].

Regarding lipid peroxidation, different outcomes were observed in the studies included in this review. In the study by Torres et al. (2016), supplementation with pequi oil, extracted through boiling and cold-pressing, led to a 32% increase in lipid peroxidation in liver homogenates, as detected by the TBARS assay. However, in other studies such as those conducted by Aguilar (2012) and Coutinho (2021), the oil was found to reduce lipid peroxidation in liver and lung samples of animals treated with pequi oil [6,12,13]. These seemingly contradictory results may be associated with genetic predispositions of inflammation markers that impact lipid metabolism, as observed in the study by Miranda-Vilela (2016), where individuals carrying the GG genotype of interleukin 6 exhibited a 2.9% higher risk of lipid peroxidation [16].

#### 3.5.6. Pequi Oil Anti-Inflammatory Effect Correlation with Physical Activity and with Genetic Polymorphisms

The practice of physical activity promotes cellular metabolism, resulting in increased oxygen consumption and the production of reactive oxygen species (ROS) [20]. These changes are crucial for optimal muscle contraction performance. However, excessive exercise can elevate ROS production beyond the capacity of endogenous antioxidants. This can lead to cellular damage due to oxidative stress. The DAMPs formed can be recognized by cells of the immune system, initiating the formation of an acute inflammatory response [57].

Pequi oil is rich in antioxidant molecules capable of attenuating oxidative stress and regulating inflammation induced by physical activity [15,16,20]. In clinical studies involving runners, the oral ingestion of 400 mg of pequi oil led to reductions in lymphocyte and neutrophil counts, as well as circulating levels of C-reactive protein. These findings, combined with the decrease in lipid peroxidation detected through the TBARs assay, provide further support for the close relationship between the antioxidant activity of pequi oil and its anti-inflammatory effect [20].

One of the complications resulting from oxidative stress is the development of cardiovascular diseases, which are associated with the common effect of intense physical activity: the elevation of blood pressure [15,58]. Monounsaturated fatty acids, such as oleic acid, which is present in large proportions in pequi oil, are linked to protective effects on the cardiovascular system, particularly in reducing blood pressure [20]. This effect was demonstrated through supplementation with pequi oil in runners, who exhibited lower blood pressure levels compared to those detected before supplementation. Furthermore, in men, total and LDL cholesterol levels were reduced, while in women, HDL levels were higher. These gender-related differences may be attributed to the influence of sex hormones on the impact of diet on the lipid profile [20].

Genetic polymorphisms contribute to changes in protein expression, consequently impacting the activity of enzymes involved in oxidative stress [59]. Additionally, the chemical compounds present in pequi oil can bind to transcription factors, alter substrate concentrations, or interact with metabolic pathways, ultimately influencing genomic stability [15]. Supplementation with pequi oil in runners demonstrated the ability to modulate genetic polymorphisms associated with predisposition to oxidative stress and the development of cardiovascular diseases [15,16].

Specifically, genotypes including haptoglobin 1S/1T/2–2, MnSOD Val9Ala, T1 null glutathione S-transferases, angiotensin I-converting enzyme ID, C-reactive protein GG, methylenetetrahydrofolate reductase C677T, and IL-6 174 G/C are linked to a higher risk of developing cardiovascular disease, evidenced by elevated levels of lipid peroxidation, LDL, VLDL, TG, total cholesterol, C-reactive protein, and platelet and leukocyte counts. Following supplementation with pequi oil in runners with these genotypes, a reduction in the aforementioned markers, as well as in diastolic and systolic blood pressures, was observed, highlighting the cardiovascular protective effect of pequi oil [15,16].

### 3.6. Nanostructured Pequi Oil

Nanotechnology is the science related to the development of materials on the nanoscale (10^−9^ m). The high interest in producing nanomaterials arises from the significant possibilities presented by the emergence of new physicochemical characteristics compared to bulk materials [60]. Particularly in biological applications, nanomaterials are an amazing tool for overcoming physiological challenges in the delivery of bioactive compounds. The production of nanophytomedicines is an emerging field that has been crucial in better understanding the mechanisms enabling the therapeutic use of plant-derived molecules with low stability, thereby increasing their biological activity [61].

Nanostructuring pequi oil is an efficient strategy for overcoming the limitation of its therapeutic use occasioned by its hydrophobic profile, which can impair its biodistribution. Nanoemulsions are characterized by having a diameter ranging between 50 and 200 nm, and are constituted by an oil phase, an aqueous phase, and a surfactant agent [62,63]. The encapsulation of pequi oil in nanoemulsions can enhance its efficiency, improving biodistribution, reducing possible adverse effects, and also shielding the oil from external factors, such as oxidation and hydrolysis, thereby preserving its bioactive compounds [13]. This was demonstrated in the study by Ombredane (2020), where pequi oil-based nanoemulsions were developed and showed a greater antitumor effect against the breast cancer 4T1 cell line compared to free pequi oil [64]. Additionally, nanocapsules containing pequi oil (*Caryocar coriaceum*) incorporated into a gel were developed in the study by Silva (2022) and demonstrated significant efficacy in the treatment of osteoarthritis, alleviating symptoms in the knee [65].

In the study conducted by Coutinho (2021), the nanoencapsulation of pequi oil in nanoemulsions (PENE) was shown to enhance the anti-inflammatory activity of the oil. Animals supplemented with 20 mg/kg of PENE exhibited an absence of leukocyte and neutrophil migration to the lungs when stimulated with LPS. Additionally, they displayed reduced levels of inflammatory cytokines such as TNF-a, IL-1b, IL-6, MCP-1, and KC. Notably, these effects were not observed in the animals treated with free pequi oil at the same dose, indicating that the PENE formulation optimized the delivery of the oil through physiological barriers directly to immune cells [13].

### 3.7. Pequi Oil Anti-Inflammatory Mechanisms of Action

One of the significant challenges in investigating the therapeutic applications of phytomedicines is elucidating the mechanisms of action related to their bioactivities. Their matrix is composed of different substances that act together, presenting synergies and antagonisms at various points in cellular metabolism [66]. Additionally, factors such as extraction methodology and seasonality directly impact chemical characterization and thus the reproducibility of molecular effects [6]. Given this context, defining the mechanism of action of phytomedicines is a complex puzzle in which the pieces come together through the investigation of their isolated substances, forming a limited view, but one that serves as a basis for understanding their therapeutic efficacy.

**Figure 4:**
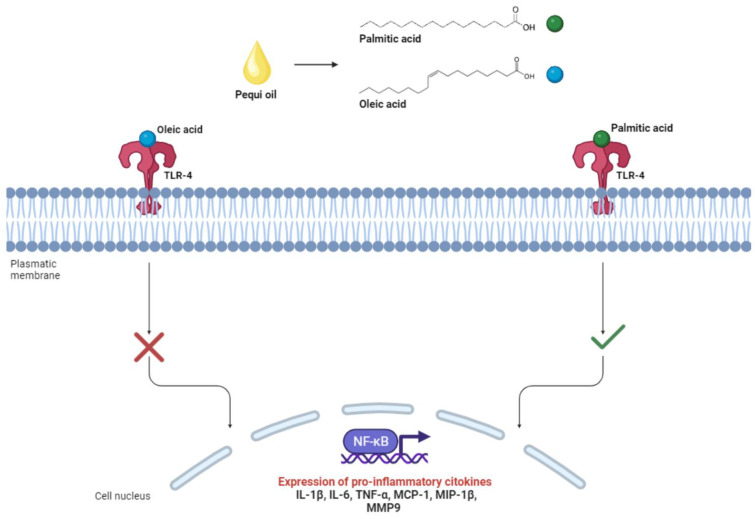
Interaction of palmitic and oleic acid with toll-like receptors 4 (TLR4) associated with the immunomodulatory responses of pequi oil.

According to the chemical characterization results of the studies included in the present review, pequi oil is primarily composed of oleic acid (~55%) and palmitic acid (40%) [6,12,13,14,15,16,18,19,20]. Both fatty acids have paradoxical effects on the inflammatory response, with palmitic acid associated with pro-inflammatory effects and oleic acid with anti-inflammatory effects [67,68]. These associations result from the interaction of these fatty acids in the cellular signaling pathway mediated by toll-like receptors 4 (TLR-4). These receptors are expressed on the membrane of immune system cells and, when activated, stimulate the nuclear factor kappa B (NF-κB) to initiate gene transcription of several pro-inflammatory mediators such as IL-1β, IL-6, TNF-α, monocyte chemoattractant protein-1 (MCP-1), macrophage inflammatory protein 1β (MIP-1β), and matrix metallopeptidase 9 (MMP9) [67]. Saturated fatty acids like palmitic acid, when bound to TRL4, stimulate the signaling pathway, resulting in inflammation activation, while monounsaturated fatty acids like oleic acid inhibit it, as illustrated in Figure 4 [67,68].

The higher concentration of oleic acid compared to palmitic acid leads to the predominance of anti-inflammatory effects through the inhibition of the TLR4 pathway. This was observed in the study by Coutinho (2020), where there was a reduction in the levels of TNF-alpha, IL-1beta, IL-6, MCP-1, and keratinocyte-derived chemokine (KC) mediators in animals treated with 20mg/kg of pequi oil-based nanoemulsions [13]. Additionally, the study by Torres (2016) showed a similar effect related to the reduction of gene expression of the inflammatory markers IL-1, TNF-alpha, IkappaB kinase-beta (IKK-beta), and transforming growth factor-beta receptor 1 (TGFR1) in animals supplemented with pequi oil extracted through an artisanal process (boiling) at a concentration of 6 mL/kg [6].

Pro-inflammatory mediators play an essential role in signaling the components of the immune system during the genesis of inflammation. A reduction in the circulating levels of these molecules impairs the migration and plasma levels of leukocytes involved in acute inflammation, primarily monocytes, macrophages, and neutrophils [23]. Different studies have demonstrated that animals treated with pequi oil have reduced leukocyte plasma levels [6,13,14,18,19]. It is worth mentioning that in the study by Coutinho (2021), 20 mg/kg of pequi oil-based nanoemulsions mitigated pulmonary leukocyte migration in mice to the same extent as treatment with 5 mg/kg of oleic acid-based nanoemulsions, providing evidence of the close relationship between oleic acid and the anti-inflammatory activity of pequi oil [13]. Similar results were also found in the clinical trial with athletes carried out by Miranda-Vilella (2009), where this effect was observed since all volunteers showed a reduction in the levels of circulating neutrophils and lymphocytes [20].

During the inflammatory response, several lipid mediators are produced. Among them, prostaglandins play crucial roles in inflammation by binding to G protein-coupled receptors, initiating an intracellular signaling cascade that, depending on the context of the inflammatory microenvironment and released cytokines, may result in the stimulation or resolution of inflammation [69]. In the study by Torres (2016), treatment with pequi oil extracted using an artisanal process was able to increase the production of prostaglandin E2 (PGE2) [6]. This same effect was observed in the study by Muller (2021) in RAW264 macrophages treated with oleic acid. It may be related to the incorporation of this fatty acid into the phospholipid composition of the cell membrane, which in turn competes with arachidonic acid at the enzymatic site of the enzyme cyclooxygenase 2 (COX-2), leading to its conversion to PGE2 [70]. Synergistically, palmitic acid was linked to increased COX-2 gene expression in RAW264.7 macrophages [71]. Within the context of the results found in the study by Torres (2016), PGE2 may exert an anti-inflammatory effect due to the reduction in the levels of leptin, IL-6, and leukotrienes 4 and 5 detected in the same groups of animals [6].

### 3.8. Toxicity Aspects of Pequi Oil

Toxicity assessment is a crucial aspect, especially when considering therapeutic applications of pequi oil. In a study conducted by Traesel (2017) to evaluate maternal, embryotoxic, and teratogenic effects, the oil was administered via gavage at concentrations ranging from 250 to 1000 mg/kg/day per group until the 15th day of gestation [72]. It exhibited no signs of toxicity and had no impact on rates of reproductive, embryonic, or fetal variables, suggesting that its consumption during pregnancy is safe for both the female and the fetus. In the same research, acute toxicity was assessed with a single dose of 2000 mg/kg/bw in female Wistar rats, and pequi oil showed low toxicity. It did not lead to fatalities, alterations in physiological habits, or changes in the animals’ health status. However, in subchronic doses of 125–1000 mg/kg for 28 days, pequi oil caused some hematological irregularities, including alterations in monocyte and lymphocyte counts, as well as higher mean corpuscular hemoglobin and mean corpuscular volume. The underlying cause of these abnormalities remains unclear, warranting further experimentation [73].

Regarding genotoxicity, studies employing the comet assay—a widely used methodology for detecting DNA damage—revealed that chronic administration of pequi oil to animals at concentrations of 125, 250, 500, and 1000 mg/kg/bw for 4 weeks resulted in a damage index and frequency of damage similar to the negative control group, indicating no significant DNA damage. Additionally, the micronucleus test demonstrated no clastogenic/aneugenic effect on the bone marrow. These findings support the results of the comet assay and indicate no genotoxic effects in male and female Wistar rats [74].

### 3.9. Relevance of the Description of the Characterization and Methodology of Extraction of Pequi Oil

The physical and chemical characteristics of pequi oil exhibit considerable variability, influenced by factors like the region of origin, soil type, climate, and harvest timing. As a result, oils extracted from different regions tend to show varying concentrations of their primary compounds [75].

Typically, pequi oil is predominantly composed of oleic acid, constituting 54 to 59% of the total, followed by palmitic acid, comprising 29% to 41% of the total fatty acids. It also contains smaller quantities of linoleic and palmitoleic acids. Furthermore, its composition is notably rich in secondary metabolites, including carotenoids, phenolic acids, lycopenes, vitamin A, vitamin C, and tocopherols [12,16,18,19,76]. However, these percentages have shown notable variations as new studies regarding the nutritional disparities of the fruit from different regions continue to emerge.

Studies indicate that pequi pulp harvested in the Goiás region contains 60% oleic acid and 28% palmitic acid, whereas pequi pulp harvested in Tocantins contains 35% and 16% of these constituents, respectively. They also reveal that the concentrations of phenolic compounds in the fruit fluctuate depending on its origin. Those harvested in Goiás have 34% fewer phenolic compounds compared to those harvested in Tocantins. Similarly, the amounts of vitamin C are approximately three times higher in fruits harvested in Minas Gerais compared to those harvested in the Goiás region [77]. Carotenoid concentrations also vary according to the region. Fruits harvested in Mato Grosso and Minas Gerais have five times more carotenoids compared to those harvested in Tocantins [77].

The concentrations of carotenoids composing pequi oil vary greatly, with some reports indicating a discrepancy of up to 6.5 times. However, many authors do not specify the region of origin of the fruit, making it difficult to attribute these variations to the location of pequi collection [6,12,75,78].

The chosen extraction method has been observed to influence the concentrations of fatty acids and bioactive compounds in pequi oil, affecting its suitability for therapeutic use [79]. Both saturated and unsaturated fatty acids, along with carotenoids, possess immunomodulatory effects and are associated with the inflammatory response due to their potent antioxidant activity, which mitigates the harmful effects of excess free radicals in the body [13]. However, carotenoids exhibit high instability owing to their extensive degree of unsaturation, rendering them more susceptible to oxidation and isomerization when exposed to abiotic factors like heat, light, and oxygen [79]. Consequently, extraction techniques involving temperature fluctuations and prolonged light exposure may yield oils with chemically oxidized unsaturated fatty acids, as well as diminished amounts of bioactive compounds such as carotenoids and phenolic acids. This directly impacts their biological properties and pharmacological potential [18,79,80,81].

It was evident that solvent extraction of pequi oil yields almost two times more carotenoids compared to mechanical extraction and boiling. However, over a period of 6 months, the concentration of carotenoids in the oil extracted by boiling remained close to the initial level, whereas in the oil extracted by solvents, it reduced to almost half, and through mechanical extraction, to a third [79]. Thus, it is of utmost importance to specify the extraction method used for pequi oil, as the technique is closely associated with the oil’s composition, preservation of its biochemical characteristics, and consequently, its biological activity.

### 3.10. Future Perspectives

As demonstrated throughout this work, pequi oil exhibits significant anti-inflammatory activity through various mechanisms. Considering inflammation’s pivotal role in the genesis of several pathological processes, the development of herbal products utilizing pequi oil emerges as a promising approach. In this context, investigating therapeutic formulations coupled with advanced technologies, such as nanostructured formulations, becomes crucial for enhancing the oil’s anti-inflammatory properties and adding value to the resulting products. Given that the oil is a native product of Brazil’s rich biodiversity, products derived from it can catalyze growth within the Brazilian bioeconomy, fostering social development in extractive communities. Nevertheless, several challenges must be addressed to scale up oil extraction and elucidate the impacts of extraction methods on the oil’s anti-inflammatory effectiveness. Additionally, there is a need for further exploration of bioactive molecules associated with this activity, particularly the secondary metabolites that have received limited attention in discussions involving pequi oil. Furthermore, standardizing marker compounds in the oil, such as oleic and palmitic acids, is essential for ensuring oil quality and promoting its utilization in pharmaceutical formulations.

### 3.11. Limitations

Some limitations were observed in the development of this systematic review. The considerable heterogeneity across intervention and evaluation models, including variations in the chemical characterizations of pequi oil, dosages, durations, routes of administration, as well as the experimental models and associated pathologies, rendered it unfeasible to conduct a meta-analysis. Furthermore, the absence of risk of bias assessment tools that encompassed all experimental models included in this review (ranging from in vitro to in vivo and clinical) restricted the analysis of the included studies to a quality analysis, which was performed using a form with questions adapted by the authors.

## 4. Conclusions

The present systematic review underscores the potential anti-inflammatory activity of pequi oil against various inflammatory pathologies. The results from the included studies indicate the oil’s ability to interact with intrinsic mechanisms of the inflammatory process, modulating gene expression and levels of both pro- and anti-inflammatory mediators, reducing oxidative stress, as well as migration of immune system cells and cardinal signs of inflammation such as edema and nociception. Moreover, the encapsulation of pequi oil in nanoemulsions has been shown to enhance its anti-inflammatory activity, overcoming potential limitations in bioavailability due to its hydrophobic nature and optimizing its interaction with biological components. Additionally, the incorporation of studies utilizing in vitro, in vivo, and clinical experimental models allowed for the observation, at varying levels of biological complexity, of either the absence or low levels of toxicity or genotoxic effects associated with pequi oil. The impacts of seasonality and extraction methodology on the chemical composition of the oil draw attention to the necessity of conducting comparative studies evaluating the therapeutic efficacy and safety of pequi oil from different sources and extraction methods to gain deeper insights into their influence on its anti-inflammatory effects. In conclusion, the demonstrated anti-inflammatory activity of pequi oil in the diverse studies encompassed in this review highlights its potential for utilization in the development of herbal medicines for complementary therapies targeting inflammatory conditions.

## Figures and Tables

**Table 1:** Summary of descriptive characteristics of the included studies.

*Study*	*Population*			*Intervention*	*Outcomes*
Author, Year/Country	Model	Fruit Species/Extraction Method	Lipid Profile Analysis	Model/Treatment Regimen	Results
Aguilar et al., 2012/Brazil [12]	In vitro: Primary culture of peritoneal macrophages obtained from female mice, C57BL/6, LDL receptor-deficient (atherosclerosis-susceptible), 6 to 8 weeks-old (n = 5) In vivo: Female mice, C57BL/6, LDL receptor-deficient (atherosclerosis-susceptible), 6 to 8 weeks-old (n = 12)	Oil from *Caryocar Brasiliense*/ Extraction method: no information	-Oleic acid: 56.98% -Palmitic acid: 34.45% -MUFA:57.89% -SFA: 36.53% -b-carotene: 18.62 mg/100 g oil -b-cryptoxantine: 17.03 mg/100 g oil -Vitamin A: 2261.9 RAE/100 g oil	In vitro: Macrophage respiratory burst: PO 7% orally in the daily diet for 2 weeks → peritoneal resident macrophage plated and stimulated or not with zymosan (1 × 10^7^ particles/50 μL) → chemiluminescence measure of ROS production over a 60-min period. In vivo: Hypercholesterolemia and atherosclerosis: PO 7% orally in the daily diet with 1.25% of cholesterol (atherogenic) for 6 weeks → Blood sample (biochemical analysis); liver (total lipids); heart and aorta (atherosclerotic lesions analysis) Antioxidant activity: PO 7% orally in the daily diet with 1.25% of cholesterol (atherogenic) for 6 weeks → hepatic lipid peroxidation (TBARS assay); plasma anti-oxidized LDL autoantibody levels (ELISA); liver antioxidant enzyme activity (SOD, and CAT). Note: Control groups received 7% soybean oil diet with 1.25% of cholesterol (atherogenic)	In vitro: ↓ ROS production (*p* < 0.05) In vivo: ↑ total cholesterol, non-HDL cholesterol and triacylglycerols (*p* < 0.05) ↑ hepatic total lipids (*p* < 0.05) ↑ in the number of advanced stage lesions in aortic root ↓ in the number of aorta atherosclerotic lesions ↓ hepatic lipid peroxidation (*p* < 0.05) No differences in antioxidant enzymes activities ↓ Anti-oxidized LDL antibody levels (*p* < 0.05)
Coutinho et al., 2020/Brazil [13]	In vivo: Male A/J mice (n not clear)	Oil from *Caryocar brasiliense*/solvent extraction (hexane)	-Oleic acid: 24.4% (NMR) and 25.9% (GC-MS)	In vivo: Pulmonary inflammation: PO, and PO-nanoemulsion (PO-NE) 20 mg/kg; oleic acid nanoemulsion (OA-NE) 5 mg/kg orally 18 and 4 h before intranasal administration of LPS → AHR analysis (lung elastance with methacholine aerosolization); Inflammatory BALF cells analysis; Lung MPO activity and cytokines levels (MCP-1, TNF-a, IL-6, IL-1b, and KC); CAT activity analysis; lung lipid peroxidation by TBARS.	In vivo: ↓ migration of leukocytes and neutrophils into the lung (PO; *p* < 0.05) No migration of leukocytes and neutrophils into the lung (PO-NE, and OA-NE similarly; *p* < 000.1) ↓ MPO activity (PO-NE, and OA-NE similarly; *p* < 0.001) ↓ TNF-a, IL-1b, IL-6, MCP-1 and KC (PO-NE; *p* < 0.001) ↓ AHR (PO, PO-NE, and OA-NE similarly; *p* < 0.001) ↑ CAT activity (PO-NE, and OA-NE similarly; *p* < 0.05) ↓ lipid peroxidation (PO-NE, and OA-NE similarly; *p* < 0.05)
Junior et al., 2020/Brazil [14]	In vivo: Male Swiss mice (n = 6); 50 days	Oil from *Caryocar brasiliense* (pulp)/cold-pressed in an “expeller” press followed by centrifugation	-Oleic acid: 56.5% -Palmitic acid: 38.11%	In vivo: -Pleurisy: PO orally 300, 700, or 1000 mg/kg 1 h before induction of pleurisy with Cg → pleural exudate was evaluated 4 h later (number of leukocytes and protein content); -Paw edema: PO orally 300, 700, or 1000 mg/kg 1 h before induction of paw edema with Cg → paw volume (evaluated 1, 2, and 4 h after Cg); mechanical hyperalgesia and cold sensitivity (3 and 4 h after Cg); -Pain model: PO orally 1000 mg/kg 1 h before induction of pain with formalin (paw) → Mechanical hyperalgesia, paw volume, and cold sensitivity (30 min after formalin); -Induced nociception: PO orally 1000 mg/kg 1 h before nociception induction with acetic acid and count of abdominal constrictions over 20 min.	In vivo: ↓ leukocyte migration (~36%) to pleural exudate (PO 1000 mg/kg; *p* < 0.05); No changes in plasma protein extravasation (pleural exudate); ↓ paw edema à PO 700 mg/kg (~ 28% after 1, 2 and 4 h); PO 1000 mg/kg (~ 60%, 42%, and 40% after 1, 2 and 4 h respectively; *p* < 0.05); ↑ Antihyperalgesic activity: PO 1000 mg/kg similar effect to Dexamethasone 1 mg/kg, *p* < 0.05; ↑ antinociceptive cold effect: PO 1000 mg/kg (~25% after 3 h; *p* < 0.05); ↓ nociception and cold sensivity induced by formalin: PO 1000 mg/kg (~95%, *p* < 0.05); ↓ nociception induced by acetic acid: PO 1000 mg/kg (~90%, *p* < 0.05);
Miranda-Vilela et al., 2009/Brazil [8]	*Clinical*: Healthy volunteers with at least a 4000 m run performance: men (n = 76) and women (49); Age: 15–67 years old	Oil from *Caryocar brasiliense* (pulp)/solvent extraction (chloroform)	-Oleic acid: 54.28%; -Palmitic acid: 41.78%; -Provitamin A: 6.26–11.5 mg/100 mg pulp -Lycopene: 1.12–2.08 mg/100 mg pulp	*Clinical* -Two races (same route and time) for each volunteer: (1) race without PO supplementation and (2) race after ingestion of 400 mg of PO capsules daily for 14 consecutive days. -Biochemical analysis (serum lipid profiles and hs-CRP); hemogram; lipid peroxidation by TBARS assay	*Clinical*: ↓ platelets and plateletocrit—total volunteers (*p* = 0.00); ↑ monocytes—total volunteers (*p* < 0.02); ↑ eosinophiles—5–19 years-old (*p* < 0.48); ↓ cholesterol and LDL, >45 years-old (mainly for men) (*p* < 0.05); ↑ HDL—total volunteers; ↑ TG and VLDL, 20–24 years-old (*p* < 0.05); ↓ hs-CRP values, 30–34 years-old (*p* < 0.05); No differences in TBARS assay Minor adverse effect in 18 volunteers (first 3 to 4 days of treatment only);
Miranda-Vilela et al., 2011/Brazil [15]	*Clinical*: Healthy volunteers with at least a 4000 m run performance: men (n = 76) and women (49); Age: 15–67 years old	Oil from *Caryocar brasiliense* (pulp)/solvent extraction (chloroform)	-Oleic acid: 54.28%; -Palmitic acid: 41.78%; -Provitamin A: 6.26–11.5 mg/100 mg pulp -Lycopene: 1.12–2.08 mg/100 mg pulp	*Clinical*: -Two races (same route and time) for each volunteer: (1) race without PO supplementation and (2) race after ingestion of 400 mg of PO capsules daily for 14 consecutive days. -Blood samples (collected after each race): Hemogram; genotyping of polymorphisms; TBARS assay; post-prandial lipid profile, and C-reactive protein.	*Clinical*: Polymorphisms that influenced PO’s responses (*p* < 0.05): -CAT, GST-M1/T1, CRP-G1059C, and MTHFR-C677T in leukogram; -Hp and MTHFR-C677T in plateletgram; -Hp, ACE, GSTT1, and MTHFR-A1298C in lipid profile; -MTHFR-A1298C in CRP levels; -Hp and MnSOD in TBARS assay; -Differences between ACE genotypes in the leukogram and total cholesterol disappeared after PO, and the same occurred e for Hp and MnSOD in TBARS assay and for MTHFR-A1298C with CRP levels.
Miranda-Vilela et al., 2016/Brazil [16]	*Clinical*: Healthy volunteers with at least a 4000 m run performance: men (n = 76) and women (49); Age: 15–67 years old	Oil from *Caryocar brasiliense* (pulp)/solvent extraction (chloroform)	-Oleic acid: 54.28%; -Palmitic acid: 41.78%; -Provitamin A: 6.26–11.5 mg/100 mg pulp -Lycopene: 1.12–2.08 mg/100 mg pulp	*Clinical*: -Two races (same route and time) for each volunteer: (1) race without PO supplementation and (2) race after ingestion of 400 mg of PO capsules daily for 14 consecutive days. -Biochemical analysis (serum ALT, AST, CK, CRP, postprandial lipid profile analyses and serum hs-CRP); hemogram; genotyping (IL-6 polymorfism → 174 G/C); lipid peroxidation (TBARS assay)	*Clinical*: Hardy–Weinberg equilibrium of IL-6–174 G/C (SNP rs1800795) genotype frequency (*p* < 0.05); ↑ GG lipid peroxidation compare to CC and GC (*p* = 0.023 and *p* = 0.041 respectively); ↑ CRP levels on CC genotype (*p* = 0.021); ↑ PDW on GC compare to GG (*p* = 0.045); ↓ LDL on CC compare to GG and GC (*p* = 0.012 and *p* = 0.03 respectively); ↓ CK and AST on the GC genotype (*p* = 0.03); No correlation between triglycerides and hs-CRP for CC genotype; ↑ 2.9 times risk of lipid peroxidation in individuals carrying GG (OR with 95% CI)
Montalvão et al., 2016/Brazil [17]	*Clinical*: Patients with systemic lupus erythematosus, men and women (n = 29) Age: 20–54 years old	Oil from *Caryocar brasiliense*/cold-pressed extraction	Not evaluated.	*Clinical*: Systemic lupus erythematosus: PO orally 400 mg for 60 consecutively days. Anthropometric measurements (BMI, AC, TS, AMC, WC, and HC); Hemogram; Biochemical analysis (urea, alkaline phosphatase, GGT, glucose, AST, ALT, complement C3, complement C4, hs-CRP, uric acid, TC, total protein and fraction (albumin and globulin), EAS, and lipid profile); DNA damage by comet assay;	*Clinical*: ↓ hs-CRP after PO treatment (*p* = 0.0161) No significant effect of pequi oil treatament on anthropometric measures, hemogram, lipid profile, EAS, biochemical analysis, and DNA damage.
Moreno et al., 2021/Brazil [18]	In vivo: Male C57BL/6 mice (n = 15)	Oil from Caryocar brasiliense/ Extraction method: no information	-Palmitic acid: 40.14 g/100 g oil -Oleic acid: 54.76 g/100 g oil -Total carotenoids: 36.16 mg g^−1^	In vivo: DSS-ulcerative colite: PO orally 280 mg homogenized in chow for 36 consecutive days → DSS-ulcerative colite was induced at the 28th treatment day → DAI (body weight loss, stools consistency and blood presence); colon histopathological analysis; Immunophenotyping of colon intraepithelial lymphocytes and splenic mononuclear cells; detection of inflammatory markers (IL-6, TNFα, IFNγ, IL-17, IL-10, and CRP); S-IgA determination.	In vivo: No significant changes in DAI ↓ crypts, and goblet cell loss (*p* < 0.05) ↓ immune cell infiltration (*p* < 0.05) ↓ colonic (*p* < 0.005), mesenteric lymph nodes, and spleen CD8+ T-cells (*p* < 0.05) No alterations on CD4+ T-cells ↑ ***γδ*** T cells at mucosa surface (*p* = 0.008) ↑ S-IgA on colon and feces (*p* < 0.05) ↓ IL-17 and CRP (*p* < 0.05) ↑ TNFα and IL-6 (*p* < 0.05)
Roll et al., 2018/Brazil [19]	In vivo: Female and male Swiss mice (n = 6); 6 to 7-old-month and 11 to 12-old-month	Oil from *Caryocar Brasiliense* (pulp)/mechanical pressure and centrifugation	-Oleic acid: 54.28%; -Palmitic acid: 41.78%; -Carotenoids: 27.75 mg/100 mg pulp -Provitamin A: 6.26–11.5 mg/100 mg pulp -Lycopene: 1.12–2.08 mg/100 mg pulp -Macronutrients (g/kg): Mg (0.114); Ca (0.97), and K (0.042); -Micronutrients (mg/kg): Fe (186.8); Mn (2.02); Zn (2.03).	In vitro: -Antioxidant activity: DPPH method (PO + DPPH reagent → 20 min → Absorbance 517 nm) In vivo: -Toxicity: PO orally 30 mg/animal/day for 15 days -Blood sample: hemogram and DNA damage (comet assay); -Bone marrow: genotoxicity (micronucleus assay)	*Antioxidant analysis*: Antioxidant activity (EC50): 26.26 mg/mL to reduce 50% of DPPH levels; In vivo: No genotoxic or clastogenic effects; ↑ lymphocytes and ↓ neutrophils + monocytes levels in 11–12 month group (female and male); *p* < 0.05; ↓ eosinophils levels in 6–7 month group (female); *p* < 0.05; ↓ WBC counts in 6–7 month group (male); *p* < 0.05; ↓ PDW counts in 11–12 month group (male); *p* < 0.05.
Torres et al., 2016/Brazil [6]	In vivo: Male Wistar rats (n = 8)	Oil from *Caryocar brasiliense*/cold-pressed and handmade (boiling) extraction	Handmade PO (HPO) Palmitic acid: 33.76% Oleic acid: 56.34% Phenolic compounds: 252.00 mg GAE/100 g Carotenoids:118.42 mg/100 g Tocopherols: 154.27 mg/kg Fitosterols: 793.32 mg/kg Cold-pressed PO (CPO) Palmitic acid: 29.48% Oleic acid: 59.99% Phenolic compounds: 113.01 mg GAE/100 g Carotenoids: 89.82 mg/100 g Tocopherols: 155.31 mg/kg Fitosterols: 808.62 mg/kg	In vivo: Hepatic toxicity: HPO 3 and 6 mL/kg; CPO 3 mL/kg by intragastric gavage for 21 consecutively days ^®^ Hepatic toxicity induced by single dose i.p. of 3 mL/kg CCl4 at day 22. Hepatic injury: -Biochemical analysis (ALT, ALP, AST, and lipid profiles); hepatic histopathological analysis; hepatic lipid analysis (total lipids and lipid peroxidation by TBARS) Antioxidant activity: -Hydrophilic and lipophilic liver antioxidant capacity; antioxidant enzymes activities (SOD, CAT, GPX, GR, GSH); Anti-inflammatory activity: -Gene expression of inflammatory markers (qRT-PCR); cytokine concentration (leptin, IL-1b, IL-6, IL-10, MCP-1, TNF-a, PPAR-g, LTB-4, LTB-5, PGE-2)	In vivo: ↓ AST and ALT levels in 30 and 38% respectively (HPO6; *p* < 0.05) ↓ AST level 67% (CPO3; *p* < 0.05) ↓ Triglycerides in 36% (CPO3; *p* < 0.05) ↑ HDL in 14, 34, and 27% (HPO3,6, and CPO3, respectively; *p* < 0.05) ↓ hepatic lesions (HPO3,6, and CPO3 similarly) ↑ lipid peroxidation in 32% (HPO6, and CPO3; *p* < 0.05) ↑ hydrophilic liver antioxidant capacity in 15 and 8% (HPO6, and CPO3, respectively; *p* < 0.05) ↑ GPX and GR activities in 38% and 36% (HPO6, and CPO3, respectively; *p* < 0.05) ↑ SOD activity in 25% (HPO6; *p* < 0.05) ↑ Hepatic GSH in 101% (CPO3; *p* < 0.05) ↓ GST gene expression (HPO3; *p* < 0.05) ↓ leptin, IL-6, LTB-4 and LTB-5 (HPO6 and CPO3 similarly; *p* < 0.05) ↓ IL-6/IL-10 ratio (HPO3 and 6 similarly; *p* < 0.05) ↑ PGE2 levels (HPO6; *p* < 0.05) ↓ TNFR gene expression (CPO3; *p* < 0.05) ↓ IL-1, TNF-a, IKK-b, TGFR1 gene expression (HPO6; *p* < 0.05) ↑ TNF-a/IL-10 gene expression ratio (CPO3; *p* < 0.05)

AC = arm circumference; ACE = angiotensin-converting enzyme; AHR = airway hyper-reactivity; AMC = arm muscle circumference; ALP = alkaline phosphatase; ALT = alanine aminotransferase; AST = aspartate aminotransferase; BALF = bronchoalveolar fluid; BMI = body mass index; CAT = catalase; CBA = cytometric bead assay; CI = confidence intervals; CK = creatine kinase; Cg = carrageenan; CRP = C-reactive protein; CRP-G1059C = C-reactive protein polymorphism; DAI—disease activity index; DPPH = 2,2-diphenyl-1-picryl-hydrazyl-hydrate; DSS = dextran sulfate sodium; EAS = abnormal elements of sediment; ELISA = enzyme-linked immunosorbent assay; GC-MS = gas chromatography–mass spectrometry; GGT = gamma glutamyl transferase; GPX = glutathione peroxidase; GR = glutathione reductase; GSH = glutathione; GST-M1/T1 = glutathione S-transferase M1 and T1; GSTT1 = glutathione S-transferase; HC = hip circumference; HDL = high-density lipoprotein; Hp = haptoglobin; hs-CRP = high-sensitivity C-reactive protein; IEL = intraepithelial lymphocytes; IFN-g = interferon-gamma; IKKb = IkappaB kinase-beta; IL = interleukins; i.p = intraperitoneal; KC = keratinocyte derived chemokine; LDL = low density lipoprotein; LPS = lipopolysaccharide; LTB = leukotriene; MCP-1 = monocyte chemoattractant protein-1; MnSOD = manganese superoxide dismutase; MPO = myeloperoxidase; MTHFR-C677T and MTHFR-A1298C = methylenetetrahydrofolate reductase polymorphisms; MUFA = monounsaturated fatty acids; NE = nanoemulsion; NMR = nuclear magnetic resonance; OA = oleic acid; OR = odds ratio; PDW = platelet distribution width; PGE-2 = prostaglandin E2; PO = pequi oil; PPAR-g = peroxisome proliferator-activated receptor g; qRT-PCR = quantitative real-time polymerase chain reaction; ROS = reactive oxygen species; SFA = saturated fatty acid; S-IgA = secretory-immunoglobulin A; SNP = single-nucleotide polymorphisms; SOD = superoxide dismutase; TBARS = thiobarbituric acid reactive substances; TG = triglycerides; TGFR1 = transforming growth factor beta receptor 1; TNF-a = tumor necrosis factor alpha; TNFR = tumor necrosis factor receptor; TS = triceps skinfold; VLDL = very low-density lipoprotein; v.s. = via subcutaneous; WBC = white blood cells; WC = waist circumference; ↑ = high; ↓ = low; → = subsequently.

## Data Availability

Data sharing is not applicable.

## Supplementary Materials

The following supporting information can be downloaded at: https://www.mdpi.com/article/10.3390/ph17010011/s1, Table S1: Database search; Table S2: Criteria of exclusion; Table S3: Quality analysis questions.
