# Supplementary material for: Anti-Inflammatory Activity of Pequi Oil (Caryocar brasiliense): A Systematic Review
Source: Pharmaceuticals (Basel). 2023 Dec 21;17(1):11. doi: 10.3390/ph17010011 (PMC10821120; doi:10.3390/ph17010011)
Supplement: Supplementary file 1 [file pharmaceuticals-17-00011-s001.zip › pharmaceuticals-2671929-supplementary/Supplementary Table S1 - Search.pdf]

Data base	String	Results	Search date
BSV regional portal	(((“Caryocar brasiliense” OR Pequi) AND (oil OR “plant oil*” OR “fatty acid” OR "vegetable oil") AND (inflammation* OR “anti-inflammatory”)))	25	31.10.2022
CINAHL	(Pequi OR "Caryocar brasiliense" OR Caryocar) AND (Oil* OR fatty acid OR "vegetable oil" OR "plant oil") AND (inflammation OR inflammatory OR "inflammatory marker" OR "antiinflammatory")	21	31.10.2022
Cochrane	(Pequi OR "Caryocar brasiliense" OR Caryocar) AND (Oil* OR fatty acid OR "vegetable oil" OR "plant oil") AND (inflammation OR inflammatory OR "inflammatory marker" OR "antiinflammatory")	6	31.10.2022
LILACS	(((“Caryocar brasiliense” OR Pequi) AND (oil OR “plant oil*” OR “fatty acid” OR "vegetable oil") AND (inflammation* OR “anti-inflammatory”)))	3	31.10.2022
Embase	('pequi oil'/exp OR pequi OR 'caryocar brasiliense'/exp) AND ('oil'/exp OR 'vegetable oil'/exp OR 'fatty acid'/exp) AND ('inflammation'/exp OR 'antiinflammatory agent'/exp OR 'inflammatory marker'/exp OR 'immune system'/exp)	31	31.10.2022
FSTA	(Pequi OR "Caryocar brasiliense" OR Caryocar) AND (Oil* OR fatty acid OR "vegetable oil" OR "plant oil") AND (inflammation OR inflammatory OR "inflammatory marker" OR "antiinflammatory")	12	31.10.2022
MEDLINE	(((“Caryocar brasiliense” OR Pequi) AND (oil OR “plant oil*” OR “fatty acid” OR "vegetable oil") AND (inflammation* OR “anti-inflammatory”)))	22	31.10.2022
ProQuest	((SU.X(Pequi)) OR "Caryocar brasiliense" OR "Pequi oil") AND (oil OR "vegetable oil" OR "fatty acid" OR "plant oil") AND (inflammation OR "inflammatory markers" OR "anti-inflammatory")<br>Refine filter “academic journals” were applied.	142	31.10.2022
PubMed	(Pequi OR "Caryocar brasiliense") AND ("Plant Oils"[Mesh]) AND ("Inflammation"[Mesh] OR "Inflammation Mediators"[Mesh] OR "Anti-Inflammatory Agents"[Mesh])	5	31.10.2022
ScienceDirect	(Pequi OR 'Caryocar brasiliense' OR 'pequi oil') AND (oil OR 'plant oil') AND (inflammation OR 'inflammatory markers' OR 'anti-inflammatory')<br>Refine filter “research articles” were applied.	64	31.10.2022
Scopus	(TITLE-ABS-KEY-AUTH (pequi OR "caryocar brasiliense" OR "Pequi oil")) AND (ALL(inflammation* OR "inflammatory response" OR "anti-inflammatory") AND (oil OR "plant oil"))	84	31.10.2022
Web of Science	ALL=((Pequi OR "Caryocar brasiliense" OR "pequi oil") AND oil* AND (inflammation* OR "inflammatory markers*" OR "anti-inflammatory"))	22	31.10.2022
