# Supplementary material for: Anti-Inflammatory Activity of Pequi Oil (Caryocar brasiliense): A Systematic Review
Source: Pharmaceuticals (Basel). 2023 Dec 21;17(1):11. doi: 10.3390/ph17010011 (PMC10821120; doi:10.3390/ph17010011)
Supplement: Supplementary file 1 [file pharmaceuticals-17-00011-s001.zip › pharmaceuticals-2671929-supplementary/Supplementary Table S2 - criteria of exclusion.pdf]

Criteria of exclusion table

**Author/ year**	**Criteria of exclusion**
Bezerra et al., 2020	3
Clael et al., 2020	3
Miranda-Vilela et al., 2014	3
Nascimento-Silva et al., 2019	4
Pires et al., 2020	3
Ribeiro et al., 2013	3
Vale et al., 2019	3
Rabbers et al., 2019	3
Gusman et al., 2015	2
Torres et al., 2020	3
Silva et al., 2022	1

Criteria of exclusion:

1- Pequi oil from different species than *Caryocar brasiliense*.

2- Pequi extract, fractionated pequi oil or other isolated compounds of *Caryocar brasiliense*.

3- Anti-inflammatory activity not evaluated in an *in vitro, in vivo* or clinical test model.

4- Review articles, book chapters, theses, letters, personal opinions, conference abstracts, and patents.
