# Supplementary material for: Anti-Inflammatory Activity of Pequi Oil (Caryocar brasiliense): A Systematic Review
Source: Pharmaceuticals (Basel). 2023 Dec 21;17(1):11. doi: 10.3390/ph17010011 (PMC10821120; doi:10.3390/ph17010011)
Supplement: Supplementary file 1 [file pharmaceuticals-17-00011-s001.zip › pharmaceuticals-2671929-supplementary/Supplementary Table S3 - Quality analysis.pdf]

Quality analysis questions

1	Are the experimental groups comparable and are control groups present?
2	Is the number of experimental units in each group clearly reported? And is it the same number (n) evaluated in statistical analyses?
3	Were pre-established criteria described for inclusion or exclusion of experimental units during the experiments or during the analyses?
4	Applicable for in vivo and clinical studies only.<br>Is there a description of any method of randomization of experimental units into treatment groups?
5	Applicable for in vivo and clinical studies only.<br>Is there a description of the application of some method of blinding the researchers, mainly those responsible the analysts of the results?
6	Were the biological variables considered for measuring the outcomes (conclusions) clearly described?
7	Have the details of the statistical analysis used in each analysis been reported?
8	Has all relevant information about the characteristics of experimental units been clearly described?
9	Are the steps of the experimental procedures and their intervals and measurements described clearly and with a level of detail that allows for replication?
10	Do the results contain data from all experiments clearly described with an indication of the statistically significant difference value (p value)?
11	Is the study abstract clear and does it contain all relevant information? As objectives, experimental design, key methods used and relevant results?
12	Does the introduction bring information that contextualizes and justifies the study?
13	Is the research question clearly stated in the study objectives?
14	Is there a description of approval of the use of cells (primary culture), animals or humans by an ethics committee and was the name of the committee reported?
15	Applicable for in vivo and clinical studies only.<br>Were the experimental conditions of experimental units allocation and supplementation clearly reported?
16	Applicable for in vivo and clinical studies only.<br>Were procedures performed to reduce stress, pain and suffering of the experimental units described?
17	Is the interpretation of the results contextualized with the objectives of the study? And were the limitations of the study described?
18	Is there a description of the results that indicates the possibility or not of translation for future experiments in other experimental models?
19	Is there information about a study protocol that was developed before the start of the experiments? If so, is there an indication of where it was published?
20	Did the study provide raw data regarding the results?
21	Is there a declaration of the presence or absence of conflicts of interest in the study?
